# Cryptococcal pneumonia: the great mimicker

**DOI:** 10.1259/bjrcr.20150358

**Published:** 2017-01-05

**Authors:** Lasya Thambidurai, R Prabhuradhan, Praveenkumar Singhvi, S Ilanchezhian, Rajoo Ramachandran, Haree Shankar

**Affiliations:** Radiology, Sri Ramachandra Medical College and Hospital, Porur, Chennai, Tamilnadu, India

## Abstract

Cryptococcal pneumonia is a fungal infection caused by *Cryptococcus neoformans* predominantly in immunosuppressed individuals and rarely in the immunocompetent population. In this study, we describe the varied radiological presentations in three patients, both immunosuppressed and immunocompetent individuals. The varied imaging presentations pose a great challenge for the radiologist and the clinician. The imaging findings mimic other diseases and it might make the diagnosis difficult purely on radiological features alone. Hence, image-guided biopsies and further evaluation are essential for confirmation of diagnosis.

Cryptococcal pneumonia is a fungal infection caused by *Cryptococcus neoformans* predominantly in immunosuppressed individuals and rarely in the immunocompetent population.^1–3^ Pulmonary cryptococcal infection is more common in the immunosuppressed population, including patients with acquired immune deficiency syndrome (AIDS), with diabetes, those on immunosuppressive therapy, etc. It is caused by the inhalation of fungal spores.^1–5^ There are two varieties of *C. neoformans*: var *neoformans *and var *gattii*. The infection is initially localized and becomes disseminated depending on the immune status of the individual. Pulmonary cryptococcosis in AIDS/immunosuppressed patients tends to manifest itself as a disseminated thoracic disease with an interstitial lung pattern and lymph node enlargement.^6,7^ In immunocompetent individuals the infection tends to be more indolent with imaging manifestations ranging from single or multiple nodular lesions or mass-like consolidation.^8–10^ The diagnosis is based on pathological findings (lymph node sampling, image-guided biopsies). The histopathological diagnosis is rendered by identifying encapsulated yeast forms within inflammatory lung tissue. Serum cryptococcal antigen test and bronchoalveolar lavage with culture can also be performed for diagnosis. Antifungal therapy is the mode of treatment; however, a few patients might need resection depending on the severity. Here, we discuss the varied radiological manifestations of cryptococcal pneumonia in three patients (immunosuppressed and immunocompetent).

## Case reviews

### Case 1

**Figure 1.**
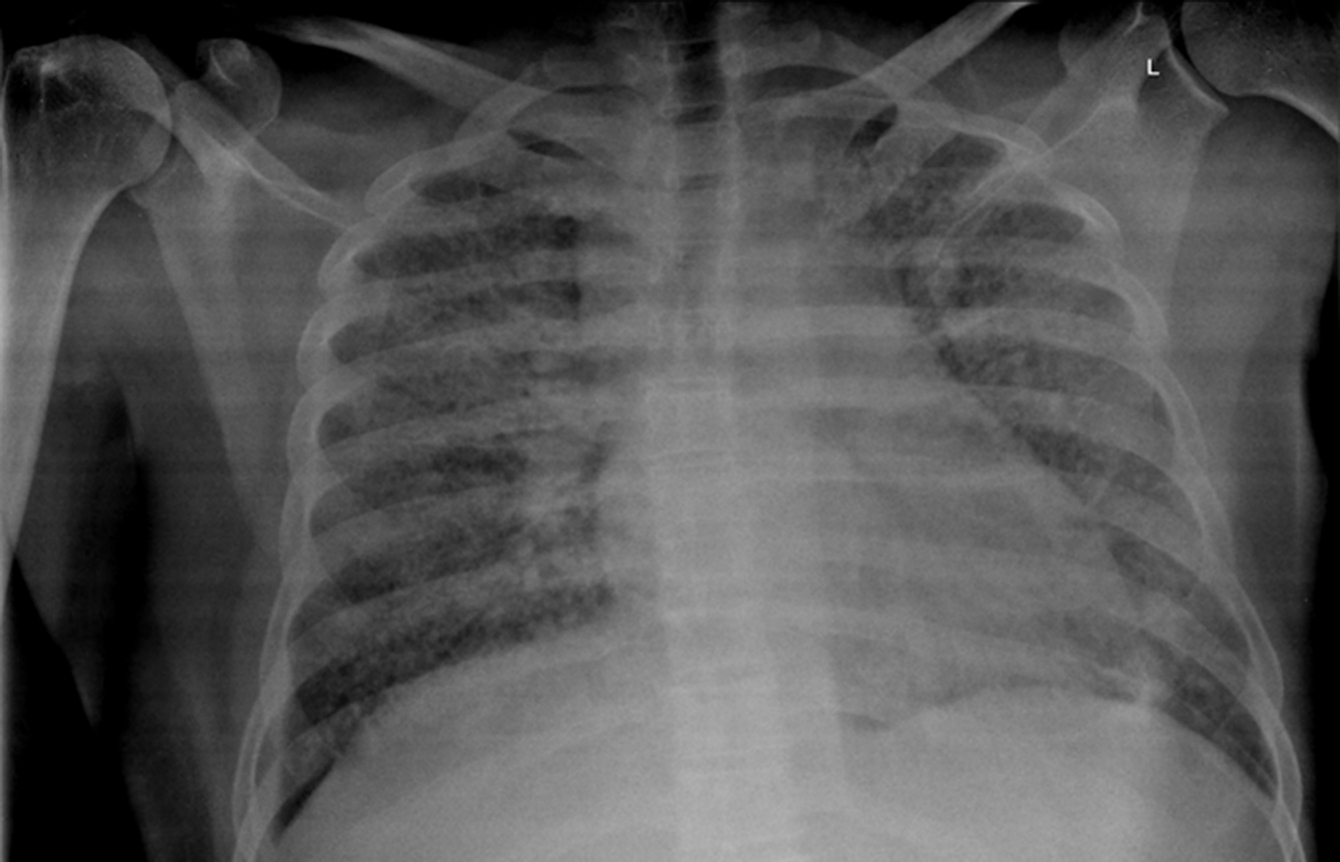
Case 1(a): Frontal chest radiograph showing multiple reticulonodular opacities in bilateral lung fields.

**Figure 2.**
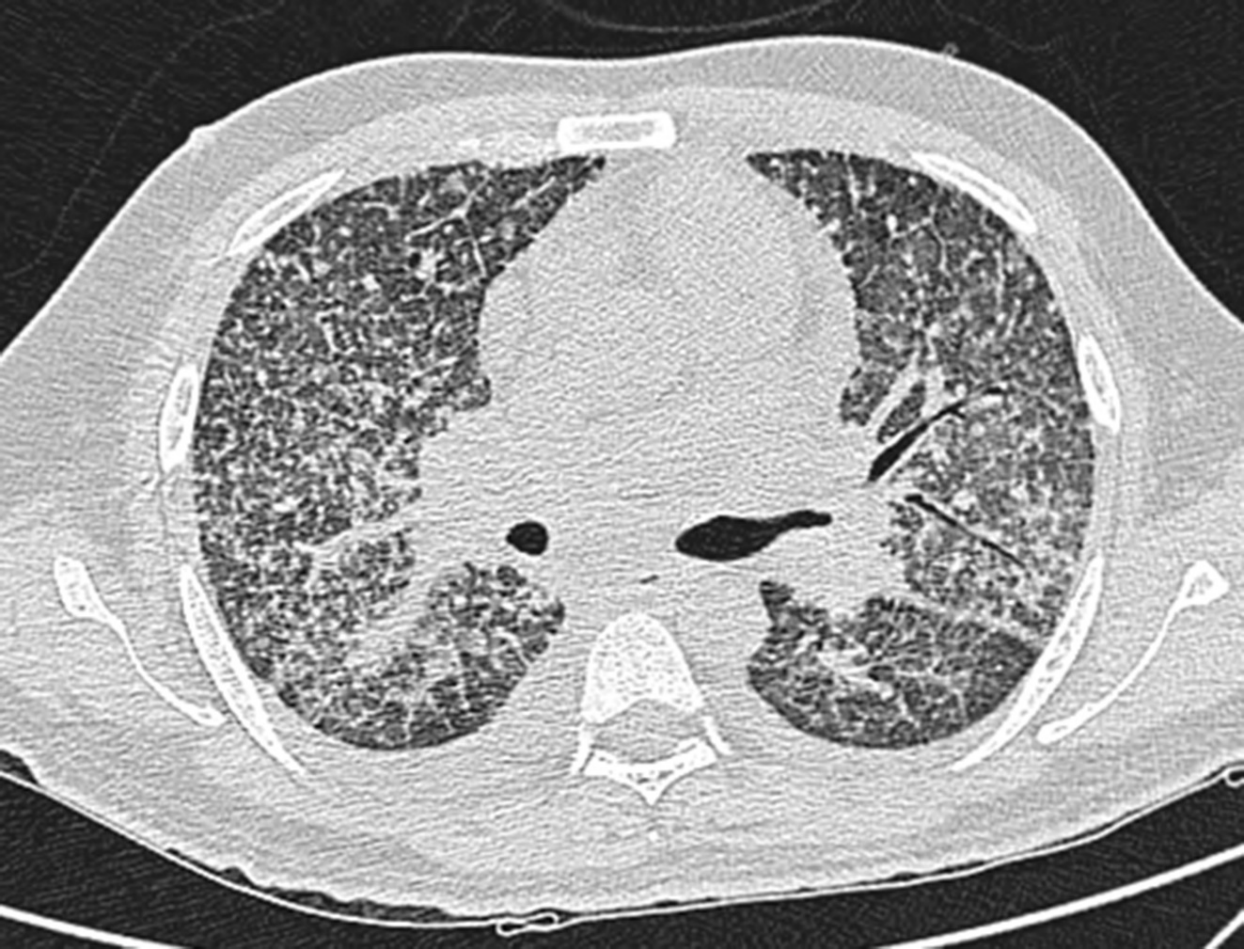
Case 1(b): CT scan of the chest in lung window shows multiple nodular opacities distributed along the peribronchovascular interstitium and subpleural regions (perilymphatic).

**Figure 3.**
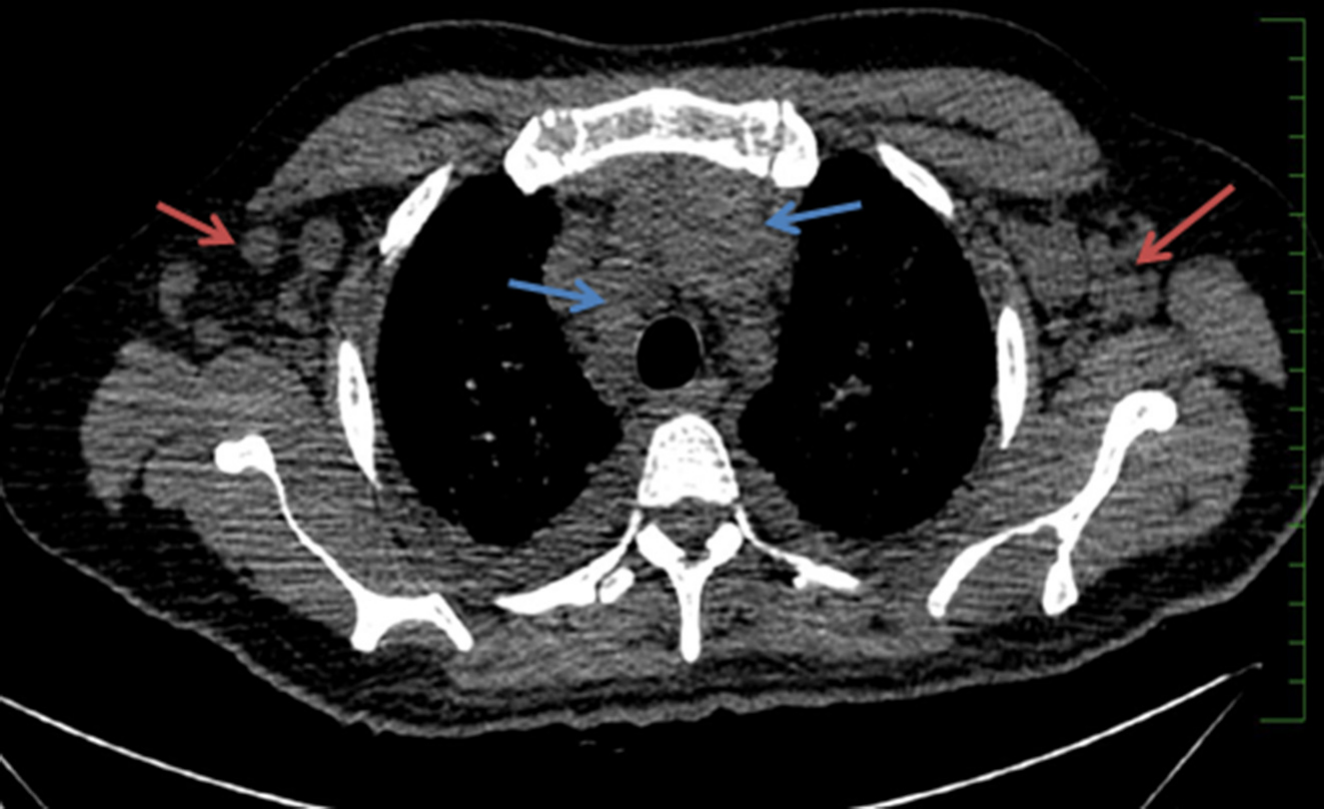
Case 1(c): CT scan of the chest in mediastinal window shows multiple enlarged axillary (red arrows) and mediastinal lymph nodes (blue arrows).

**Figure 4.**
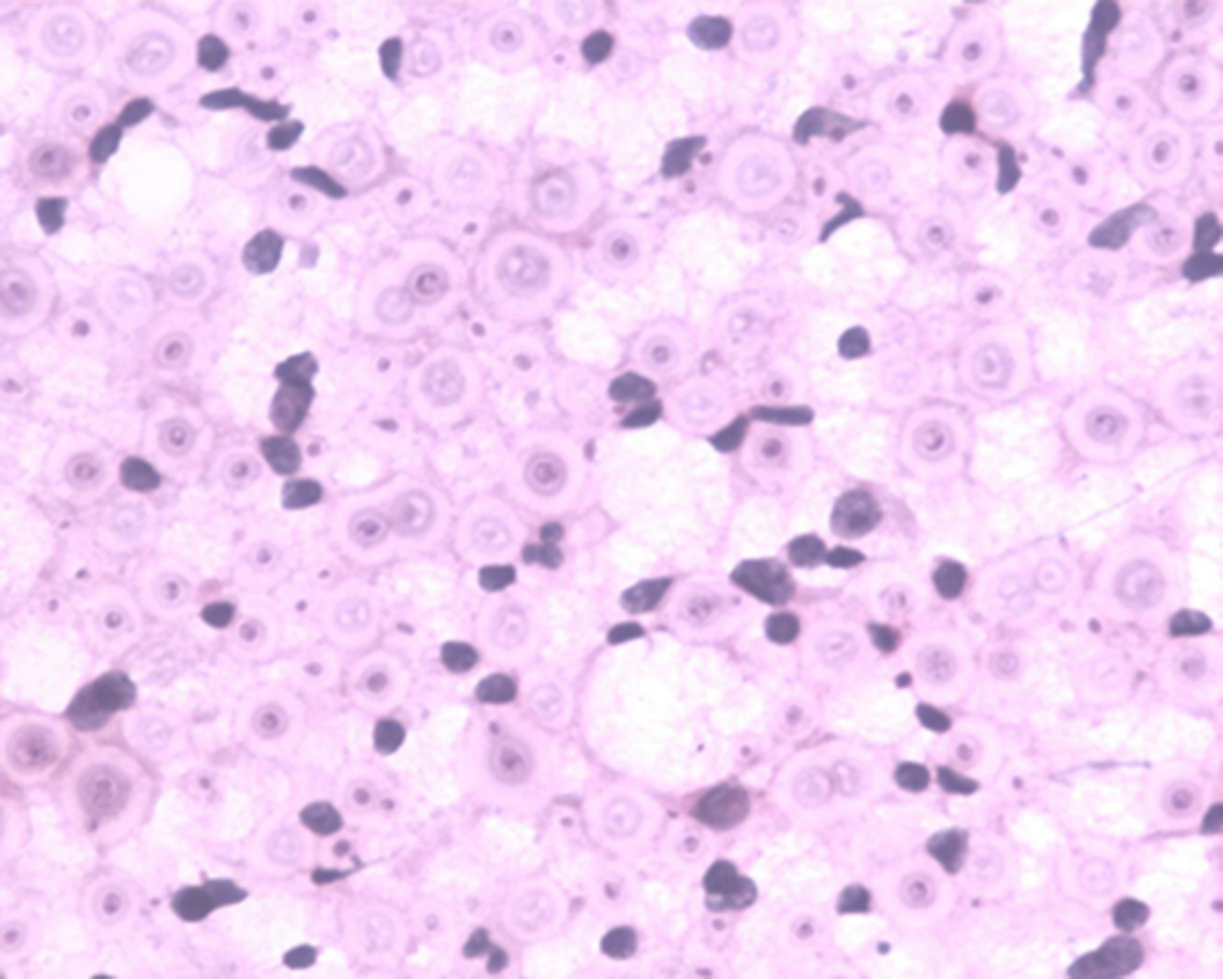
Case 1(d): Histopathological examination of the axillary lymph nodes shows evidence of cryptoccoccal infection (capsulated organisms) (magnified view).

A 40 year-old immunosuppressed male diagnosed with AIDS presented with complaints of cough and fever. Initial chest X-ray showed nodular opacities in bilateral lung fields (Figure 1). Plain CT scan showed multiple small (<2 mm) nodules distributed in the bilateral lung fields. On further analysis of the distribution of nodules they were predominantly seen along the peribronchovascular interstitium and subpleural (perilymphatic) regions (Figure 2). Multiple enlarged lymph nodes were seen in the mediastinum and axillary regions (Figure 3). Image-guided fine needle aspiration cytology of the axillary node was performed and the diagnosis of cryptococcal infection was made (Figure 4) based on the histopathological findings, which demonstrated capsulated organisms. The patient was started on antifungal therapy and dramatically improved clinically.

### Case 2

**Figure 5.**
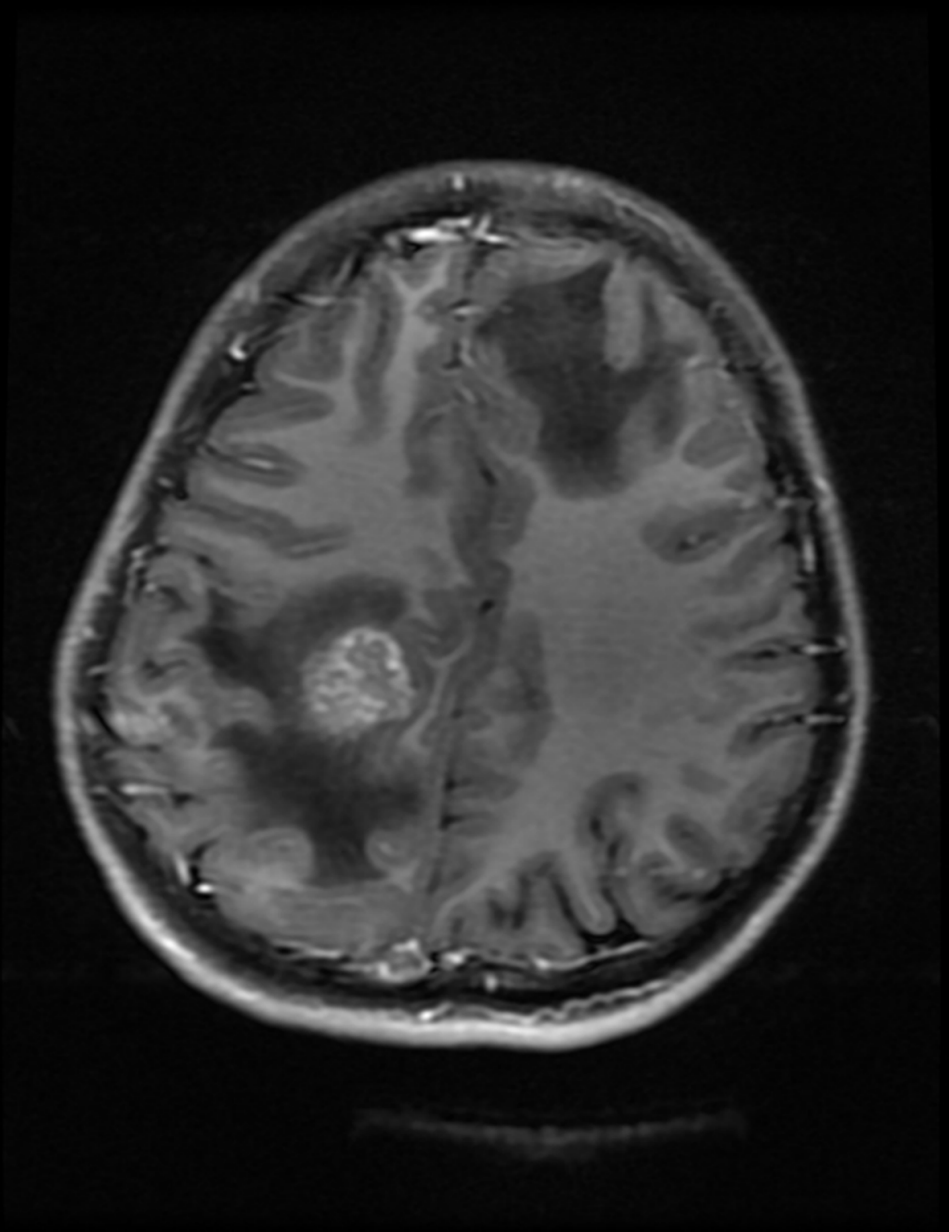
Case 2(a): Axial *T*_1_ weighted post-contrast image of brain shows ring-enhancing lesion with surrounding oedema in the right parietal lobe.

A 30-year-old immunocompetent female patient came with complaints of fever and headache for 1 month associated with vomiting. The patient was evaluated for immune-suppressed states such as diabetes mellitus, AIDS and hepatitis B and C virus infections, and was negative. In view of the fever and headache, a provisional diagnosis of meningoencephalitis was made and CT brain scan with contrast was ordered. CT brain scan with contrast showed multiple ring-enhancing lesions. MRI brain spectroscopy was performed, which showed ring-enhancing lesions with surrounding oedema (Figure 5). These lesions were reported to be tuberculomas based on the imaging findings and MRI spectroscopy findings.

**Figure 6.**
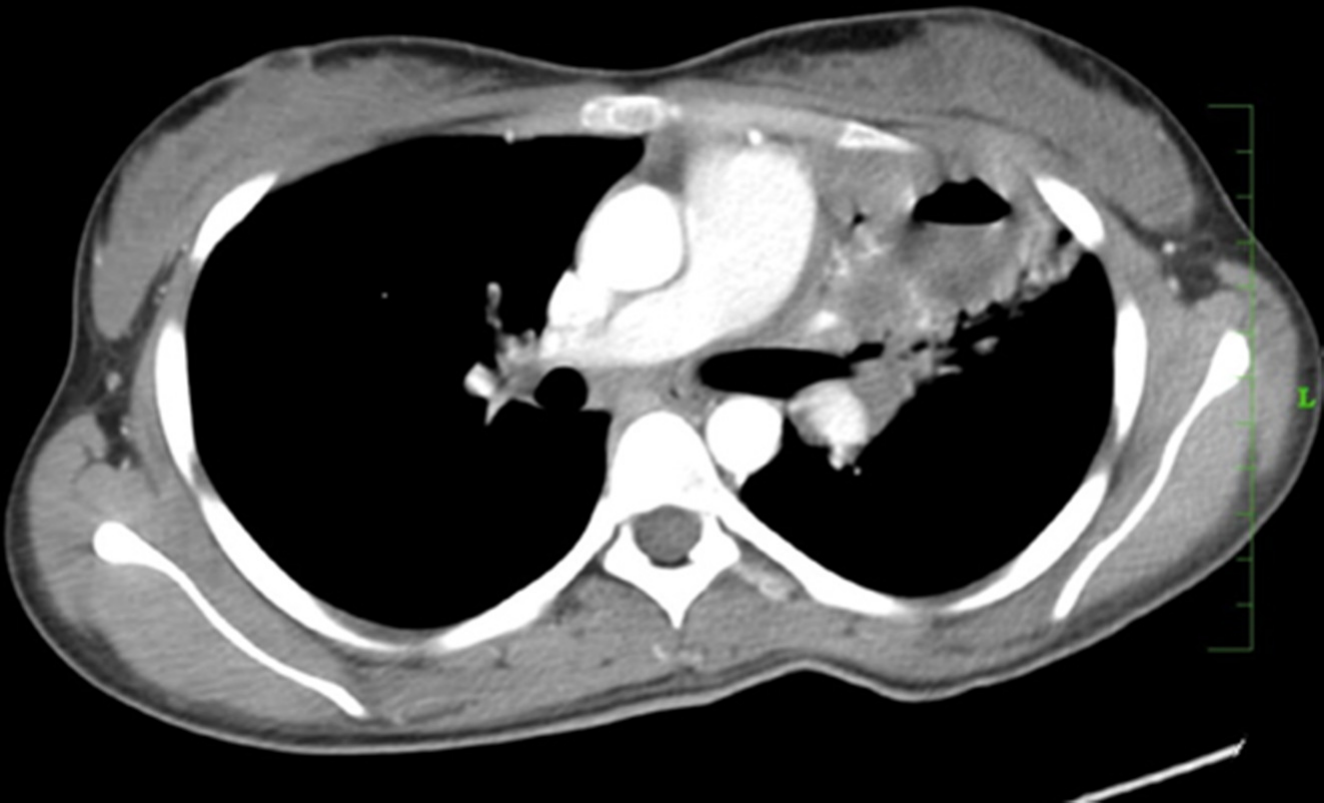
Case 2(b): Axial post-contrast CT image of the chest in mediastinal window shows cavitating consolidation in the left upper lobe with extension into the hilum.

**Figure 7.**
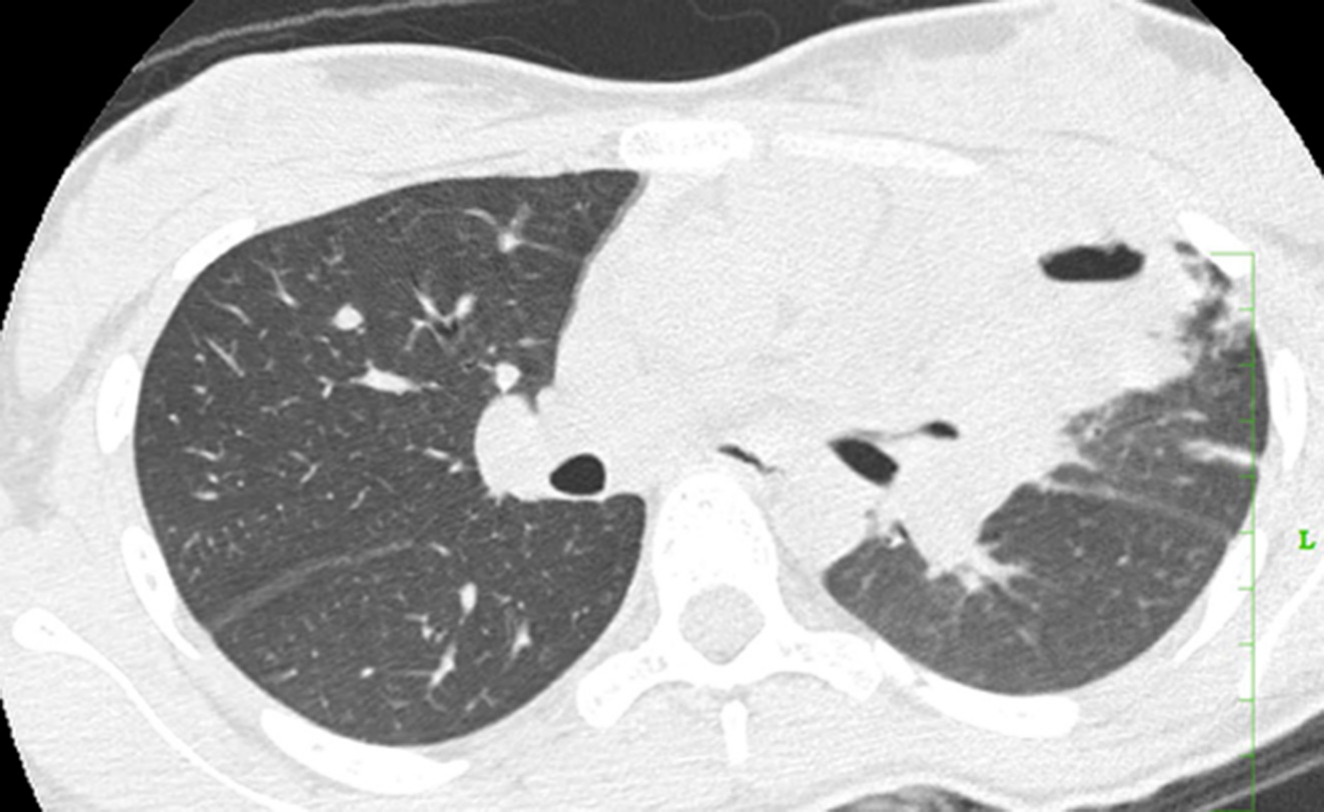
Case 2(c): Axial CT image of the chest in lung window shows cavitating consolidation in the left upper lobe.

**Figure 8.**
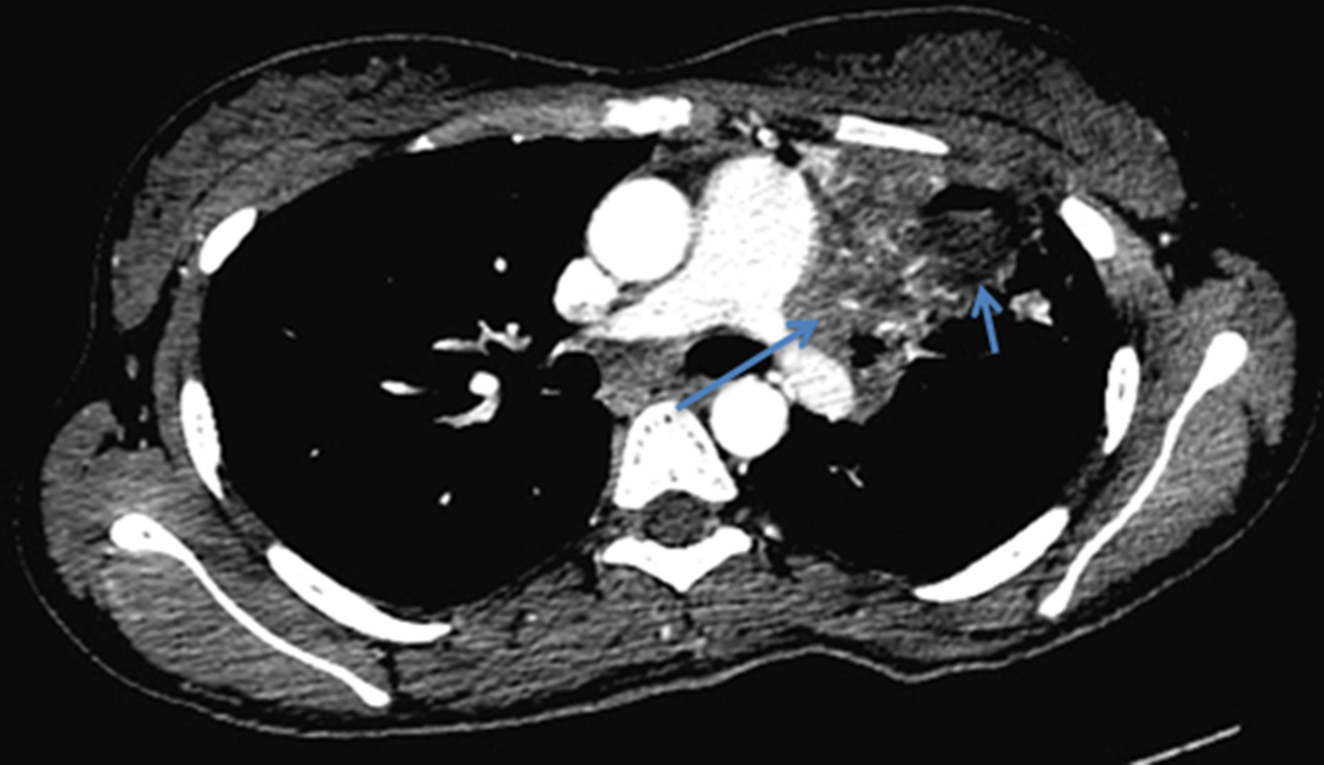
Case 2(d): Axial post-contrast CT image of the chest in mediastinal window shows cavitating consolidation (short arrow) in the left upper lobe with extension into the hilum and narrowing of the hilar structures (long arrow).

CT thorax scan was performed with administration of intravenous contrast, which showed consolidation with cavitation in the left upper lobe (Figures 6 and 7). There was associated soft tissue extension along the left hilum encasing and narrowing the left pulmonary vessels and left bronchus with features of fibrosing mediastinitis (Figure 8). Enlarged mediastinal lymph nodes were seen. In view of the brain lesions and the lung findings the possibility of tuberculosis was raised. CT-guided biopsy of the thick-walled cavity was performed and histopathological and microbiological findings showed features of cryptococcus pneumonia. The patient was started on antifungal therapy and he improved symptomatically.

### Case 3

**Figure 9.**
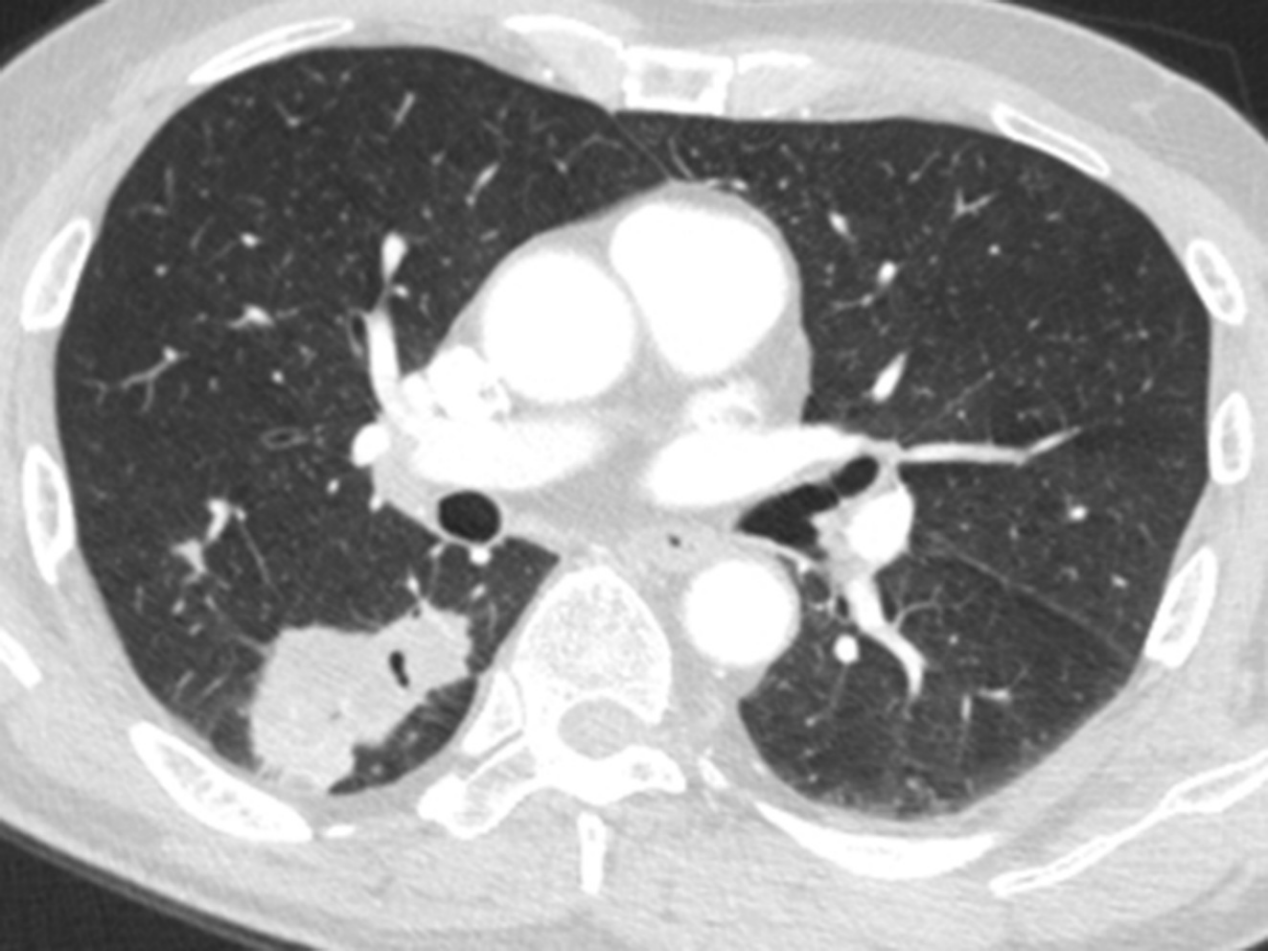
Case 3: Axial CT image of the thorax in lung window shows a mass with irregular margins in the right lower lobe.

A 5-year-old boy on immunosuppressive therapy after bone marrow transplantation presented with cough. In view of the persistent symptoms, CT thorax scan (plain) was performed. CT thorax scan showed a mass in the right lower lobe with mild irregular margins simulating a malignant mass lesion (Figure 9). In view of the suspicious features, CT-guided biopsy was performed. A diagnosis of cryptococcal infection was made based on the histopathological features. The patient was started on antifungal therapy but succumbed owing to widespread sepsis.

## Discussion

Cryptococcal infections are common in immunosuppressed individuals and relatively rare in the immunocompetent population. *Cryptococcus neoformans* is the causative organism and central nervous system is the most commonly affected organ. Pulmonary cryptococcal infection is acquired via inhalation of spores. Pulmonary cryptococcal infection occurs in both immunosuppressed and immunocompetent populations, the former being more common.^1–5^

Pulmonary cryptococcal infection has a variety of manifestations from lung nodules to mass-like appearance. The most common radiographic manifestations of pulmonary cryptococcosis consist of single or multiple pulmonary nodules, segmental or lobar consolidation or a reticulonodular pattern of opacities.^11,12^ Associated features include cavitation, lymphadenopathy and pleural effusion.^13^

In the first case, the cross-sectional imaging findings were extensive mediastinal and axillary lymphadenopathy. In the lung, there were multiple nodules scattered in bilateral lung fields, predominantly along the bronchovascular bundles, and a perilymphatic distribution. The differentials for perilymphatic nodules include sarcoidosis, silicosis, coal worker’s pneumoconiosis, lymphangitis carcinomatosis, lymphocytic interstitial pneumonia and rarely infections such as tuberculosis and atypical infections such as cryptococcosis.

The patient was immunocompromised (retropositive), and since tuberculosis is more rampant in our country Koch’s infection was the initial diagnosis. But later, fine needle aspiration cytology proved otherwise and showed evidence of multiple cryptococcomas. The patient was then treated with antifungal medication. Perilymphatic nodules in cryptococcal infection are a rare manifestation. According to the study done by Chang et al,^14^ among the nodular pattern the perilymphatic distribution of nodules was the least common pattern. The common differential diagnosis for miliary nodules in the setting of immunosuppressed status would be tuberculosis. Common infections in the background of immunosuppressed status are tuberculosis and other mycobacterial infections and fungal infections including *Pneumocystis carnii*.

The immunocompetent patient in our study presented with cavitating pneumonia, associated fibrosing mediastinitis and mediastinal lymphadenopathy. The patient also had ring-enhancing lesions as revealed by CT and MRI scan. These findings were in favour of an infective aetiology such as tuberculosis in view of the lung and brain findings. CT-guided biopsy was performed and it revealed a cryptococcal aetiology. Cavitation was considered a rare finding in immunocompetent patients according to the study by Khoury et al,^15^ and was a common feature in immunosuppressed individuals, although according to the study by Fox and Muller et al,^9^ Lindell et al^16^ and Chang et al, shows that cavitation was seen in few immunocompetent patients. Cryptococcal pneumonia with fibrosing mediastinitis is very rare and is reported less often.^17^ Such widespread cryptococcal infection involving the central nervous system and lung is rare in an immunocompetent individual.^17^ Chronic indolent lung lesions of various patterns including pulmonary cryptococcosis, tuberculosis, actinomycosis, and semi-invasive aspergillosis should be considered for the differential diagnosis of chronic pneumonia.

The third patient was immunosuppressed, post bone marrow transplantation status, with imaging findings of a mass in the right lower lobe with irregular margins. CT-guided biopsy showed features of cryptococcal pneumonia. Common differential diagnosis for this imaging feature is a neoplastic lesion and at times it might be difficult to differentiate based on imaging alone and hence tissue diagnosis is essential.

There are various studies on the imaging manifestations of pulmonary cryptococcosis. The patterns observed in immunocompetent and immunocompromised individuals show few differences. The most common patterns observed in immunocompetent population^15^ are solitary or multiple pulmonary nodules or masses with consolidation and lymphadenopathy. Pleural abnormality is rare. In contrast, the immunosuppressed individuals present with more widespread disease,^18^ with areas of cavitation and associated pleural abnormalities as described in the study by Chang et al^14^.

Cryptococcal pneumonia has a variety of radiological presentations and hence it is necessary for the radiologist to be aware of the imaging manifestations and help in early diagnosis.

## Conclusions

Although there are a few studies in literature based on the imaging findings of cryptococcal pneumonia in immunosuppressed and immunocompetent individuals, the varied imaging presentations pose a great challenge for the radiologist and the clinician. In countries such as India where tuberculosis is more prevalent, the similarity of imaging findings might make diagnosis based purely on radiological features alone difficult. Hence, image-guided biopsies and further evaluation are essential for confirmation of diagnosis.

## Learning points

1. Cryptococcal pneumonia is one of the commonly misdiagnosed entities.
2. Cryptococcal pneumonia has varied radiological presentations from a solitary nodule, disseminated miliary/reticulonodular pattern to a mass-like appearance mimicking malignancy.
3. Cryptococcal pneumonia should be in the differential diagnosis in an immunocompromised individual along with *Pneumocystis carnii* pneumonia and mycobacterial infections for pulmonary infections.
4. Although cryptococcal pneumonia is more common in the immunosuppressed individuals, it can present in an atypical pattern in immunocompetent hosts as well. Hence it is necessary for the physicians and radiologists to be aware of the varied imaging findings and tissue sampling for histopathological confirmation before commencing treatment.

## Consent

Informed consent was obtained to publish it in our case series.
